# Supplementary material for: Resolving the full spectrum of human genome variation using Linked-Reads
Source: Genome Res. 2019 Apr;29(4):635–45. doi: 10.1101/gr.234443.118 (PMC6442396; doi:10.1101/gr.234443.118)
Supplement: Supplemental Material [file supp_gr.234443.118_Supplemental_longranger-2.2.zip › longranger-2.2.tar.bz2!/lr2.2-public/lib/stan/cmdstan/stan/doc/api/html/rep__array_8hpp_source.html]

Stan: src/stan/math/rep\_array.hpp Source File


|  |  |
| --- | --- |
|  | Stan  2.5.0  probability, sampling & optimization |


- Main Page
- Related Pages
- Namespaces
- Classes
- Files

- File List
- File Members

All Classes Namespaces Files Functions Variables Typedefs Enumerations Enumerator Friends Macros Pages

- src
- stan
- math

rep\_array.hpp

Go to the documentation of this file.

1 #ifndef STAN\_\_MATH\_\_REP\_ARRAY\_HPP

2 #define STAN\_\_MATH\_\_REP\_ARRAY\_HPP

3

4 #include <vector>

5

6 #include <stan/math/error\_handling/check\_nonnegative.hpp>

7

8 namespace stan {

9

10  namespace math {

11

12  template <typename T>

13  inline std::vector<T>

14  rep\_array(const T& x, int n) {

15  check\_nonnegative("rep\_array(%1%)", n,"n", (double\*)0);

16  return std::vector<T>(n,x);

17  }

18

19  template <typename T>

20  inline std::vector<std::vector<T> >

21  rep\_array(const T& x, int m, int n) {

22  using std::vector;

23  check\_nonnegative("rep\_array(%1%)", m,"rows", (double\*)0);

24  check\_nonnegative("rep\_array(%1%)", n,"cols", (double\*)0);

25  return vector<vector<T> >(m, vector<T>(n, x));

26  }

27

28  template <typename T>

29  inline std::vector<std::vector<std::vector<T> > >

30  rep\_array(const T& x, int k, int m, int n) {

31  using std::vector;

32  check\_nonnegative("rep\_array(%1%)", k,"shelfs", (double\*)0);

33  check\_nonnegative("rep\_array(%1%)", m,"rows", (double\*)0);

34  check\_nonnegative("rep\_array(%1%)", n,"cols", (double\*)0);

35  return vector<vector<vector<T> > >(k,

36  vector<vector<T> >(m,

37  vector<T>(n, x)));

38  }

39

40  }

41 }

42

43 #endif

stan::math::rep\_array

std::vector< T > rep\_array(const T &x, int n)

**Definition:** rep\_array.hpp:14

check\_nonnegative.hpp

stan::math::check\_nonnegative

bool check\_nonnegative(const char \*function, const T\_y &y, const char \*name, T\_result \*result)

**Definition:** check\_nonnegative.hpp:52


---

[ Stan Home Page ]

*© 2011–2014,
Stan Development Team.*
